# Supplementary material for: Evolving Models of Pavlovian Conditioning: Cerebellar Cortical Dynamics in Awake Behaving Mice
Source: Cell Rep. 2015 Nov 19;13(9):1977–88. doi: 10.1016/j.celrep.2015.10.057 (PMC4674627; doi:10.1016/j.celrep.2015.10.057)
Supplement: Document S1. Figures S1 and S2 and Tables S1 and S2 [file mmc1.pdf]

Cell Reports

Supplemental Information

Evolving Models of Pavlovian Conditioning: Cerebellar Cortical Dynamics in Awake Behaving Mice

Michiel M. ten Brinke, Henk-Jan Boele, Jochen K. Spanke, Jan-Willem Potters, Katja Kornysheva, Peer Wulff, Anna C.H.G. IJpelaar, Sebastiaan K.E. Koekkoek, and Chris I. De Zeeuw

Figure S1

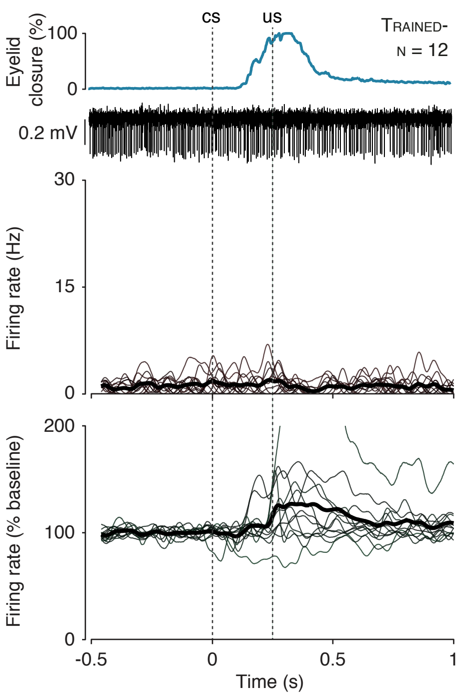

Figure S1. Purkinje cells without US-related complex spikes, Related to Figure 1. Example behavioral and spike trace in the top panel, complex spike density functions for twelve Purkinje cells in the middle panel, simple spike density functions in the bottom panel. These cells did not show US-complex spike responses and were recorded in trained mice. Seven cells showed no significant simple spike modulation, one showed suppressive modulation, and three showed facilitation in the ISI. This facilitation was not observed in cells with a clear complex spike response to the US.

Figure S2

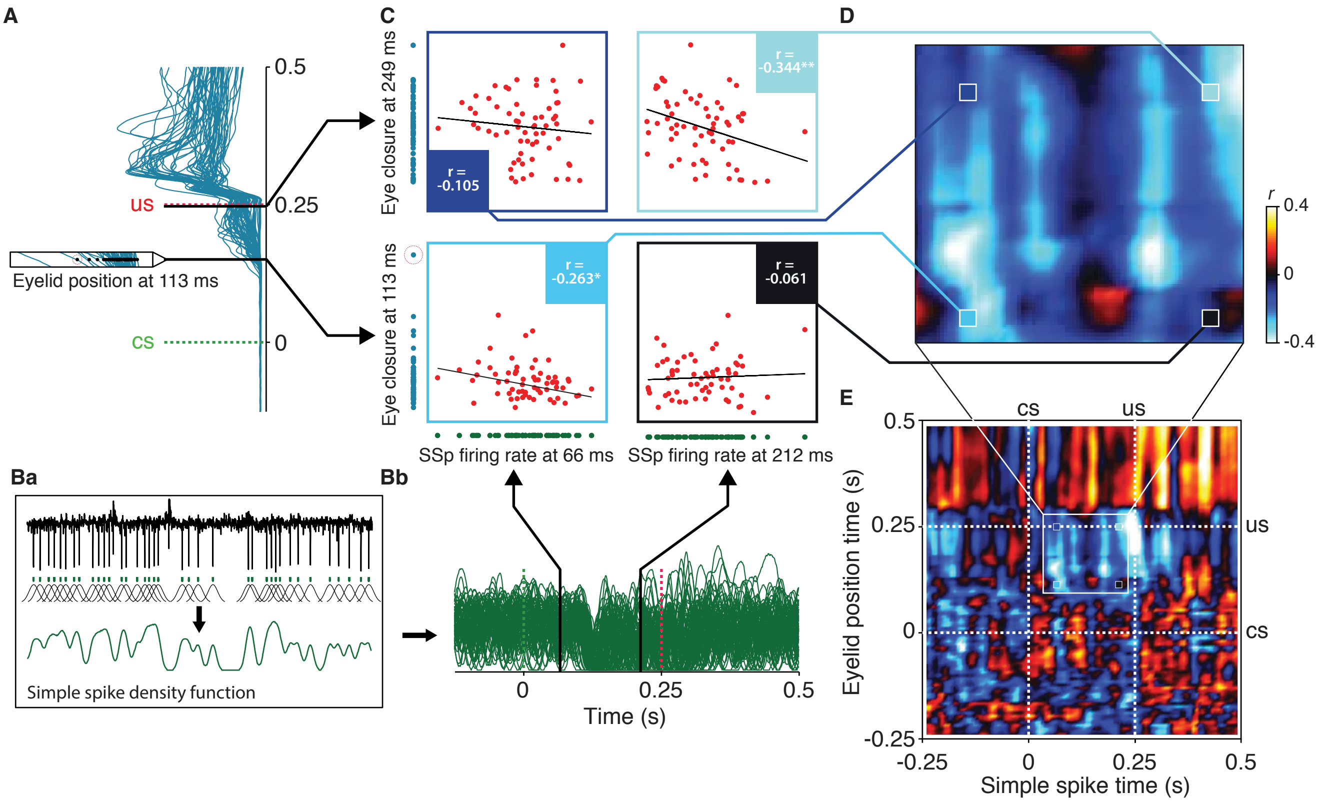

Figure S2. Construction of the simple spike-eyelid position correlation matrix, Related to Figure 2. (A) The y-axis of the eventual correlation matrix, E, denotes the time at which eyelid position data is taken. Here, eyelid traces for one recording are shown, rotated 90° counterclockwise. In the example at 113 ms after CS onset, the magnified window shows for each trial 10 data points, of which the means (black dots, red dashed circle indicates an outlier) are used for a correlation with spike activity. The same approach is taken to get eyelid position data at 249 ms. (Ba) To create a spike density function for each trial, Purkinje cell simple spike timestamps are convolved with a 41 ms wide Gaussian kernel, shown overlaid in black. The summated green trace provides a continuous measure of simple spike density. (Bb) The x-axis of the eventual correlation matrix, E, denotes the time at which simple spike activity is taken. Here, spike density traces for all trials of the same recording as in A are shown. Using the same approach outlined in A, 10-datapoint means are extracted for each trial at two example locations, here 66 and 212 ms after CS onset. (C) Between-trial correlations of eyelid position and simple spike activity data extracted at the example time-points. Simple spike activity at 66 ms correlates more strongly to eyelid position at 113 ms, than at 249 ms. Conversely, spike activity at 212 ms relates better to eyelid position at 249 ms than at 113 ms. If these correlations were done across different recordings, their trial data would be standardized before combining them. (D) The r-values for each of the example correlations translate to a color and find their way into the correlation matrix at the appropriate time points on the x- (spike) and y- (eyelid position) axis. (E) The resultant full correlation matrix shows the temporal dynamics of the correlation between simple spike activity and eyelid position. The current cell shows negative correlation throughout the ISI, with a minor early focal point, and a major late focal point.

Table S1. Summary of linear mixed-effects models, Related to Experimental Procedures.

**A.** For outcome variable **% Simple spike suppression** with random intercepts for **Purkinje cell ID**:				
**Fixed effect**	**Coefficient**	**F-test**	**P**	**Power***
Intercept	22.39 ± 2.53	$F_{1,972}$ = 78.03	<0.0001	**-**
CR (yes/no)	4.4 ± 1.14	$F_{1,972}$ = 14.87	0.00012	96.4%
**B.** For outcome variable **CR amplitude (% eyelid closure)** with random intercepts and slopes for **Purkinje cell ID**:				
**Fixed effect**	**Coefficient**	**F-test**	**P**	**Power**
Intercept	32.69 ± 2.47	$F_{1,699}$ = 174.51	<0.0001	-
% Simple spike suppression	0.334 ± 0.084	$F_{1,699}$ = 15.82	<0.0001	99%
**C.** For outcome variable **% Simple spike suppression** with random intercepts for **Purkinje cell ID**:				
**Fixed effect**	**Coefficient**	**F-test**	**P**	**Power**
Intercept	23.78 ± 2.3	$F_{1,972}$ = 107.3	<0.0001	-
CS-Complex spike (yes/no)	4.57 ± 1.14	$F_{1,972}$ = 16.2	<0.0001	86.2%
**D.** For outcome variable **CR amplitude (% eyelid closure)** with random intercepts for **Purkinje cell ID**:				
**Fixed effect**	**Coefficient**	**F-test**	**P**	**Power**
Intercept	37.98 ± 3.33	$F_{1,352}$ = 130.04	<0.0001	**-**
CS-Complex spike (yes/no)	5.20 ± 2.22	$F_{1,352}$ = 5.52	0.0194	83.8%
**E.** For outcome variable **CR amplitude (% eyelid closure)** with random intercepts for **Purkinje cell ID:**				
**Fixed effect**	**Coefficient**	**F-test**	**P**	**Power**
Intercept	65.28 ± 8.82	$F_{1,245}$ = 61.09	<0.0001	-
CS-Complex spike latency (ms)	-0.26 ± 0.10	$F_{1,245}$ = 8.74	0.0034	93.8%
**F.** For outcome variable **CS-Complex spike latency (ms)** with random intercepts for **Purkinje cell ID**:				
**Fixed effect**	**Coefficient**	**F-test**	**P**	**Power**
Intercept	91.47 ± 3.31	$F_{1,345}$ = 764.35	<0.0001	-
CR (yes/no)	-5.16 ± 1.29	$F_{1,345}$ = 15.99	<0.0001	98.2%
**G.** For outcome variable **CR amplitude (% eyelid closure)** with random intercepts for **Purkinje cell ID:**				
**Fixed effect**	**Coefficient**	**F-test**	**P**	**Power**
Intercept	29.24 ± 3.79	$F_{1,351}$ = 59.69	<0.0001	-
% Simple spike suppression	0.29 ± 0.06	$F_{1,351}$ = 24.48	<0.0001	99.8%
CS-Complex spike (yes/no)	3.08 ± 2.19	$F_{1,351}$ = 1.98	0.1604	-
**H.** For outcome variable **CR amplitude (% eyelid closure)** with random intercepts for **Purkinje cell ID**:				
**Fixed effect**	**Coefficient**	**F-test**	**P**	**Power**
Intercept	55.02 ± 8.8	$F_{1,246}$ = 38.83	<0.0001	-
% Simple spike suppression	0.24 ± 0.07	$F_{1,246}$ = 12.94	0.0004	94.6%
CS-Complex spike latency (ms)	-0.24 ± 0.09	$F_{1,246}$ = 7.65	0.0061	79.4%

Table S2. Summary of molecular layer interneuron recordings, Related to Figure 4.

Cell #	Hz	CV	CV2	# Valid Trials	Mean CR amplitude (%)	Mean CR onset (ms)	% CRs	% Spike facilitation in ISI	ISI Hz x CR amplitude r-value
1	6.6	0.69	0.66	25	68.1	105.6	88	673**	0.29
2	23.2	0.84	0.31	34	46.1	82.8	97.1	104**	-0.59***
3	17.8	0.84	0.58	8	53.5	91.3	100	78**	-0.24
4	8.4	0.74	0.62	10	30.7	104	100	233**	0.19
5	7.9	1.52	0.91	85	58.6	127.5	95.3	175**	0.34**
6	36.8	1.53	1.00	17	61.9	83.7	94.1	279**	0.73**
7	11.4	1.26	0.65	28	58.3	99.5	96.4	894**	-0.55**
8	26.3	0.82	0.53	15	65.6	98.2	100	192**	0.47
9	8.5	1.45	0.99	15	74.8	96.6	100	1048**	0.12
10	24.5	0.89	0.41	29	53.2	57.1	93.1	174**	0.73**
11	20.9	0.69	0.61	73	33.6	149.4	34.2	98**	0.41*
12	22.0	0.70	0.58	40	32.5	85.2	87.5	79**	0.63*
13	41.8	1.37	0.76	47	25.3	94.2	78.7	150**	0.40***
Mean	19.7	1.03	0.66	33	50.3	98.1	89.6	321.3	0.23

* $p < 0.05$, ** $p < 0.005$, *** $p < 0.001$
