# Supplementary material for: Evolving Models of Pavlovian Conditioning: Cerebellar Cortical Dynamics in Awake Behaving Mice
Source: Cell Rep. 2015 Nov 19;13(9):1977–88. doi: 10.1016/j.celrep.2015.10.057 (PMC4674627; doi:10.1016/j.celrep.2015.10.057)
Supplement: Document S2. Article plus Supplemental Information [file mmc2.pdf]

Cell Reports

Article

Evolving Models of Pavlovian Conditioning: Cerebellar Cortical Dynamics in Awake Behaving Mice

Graphical Abstract

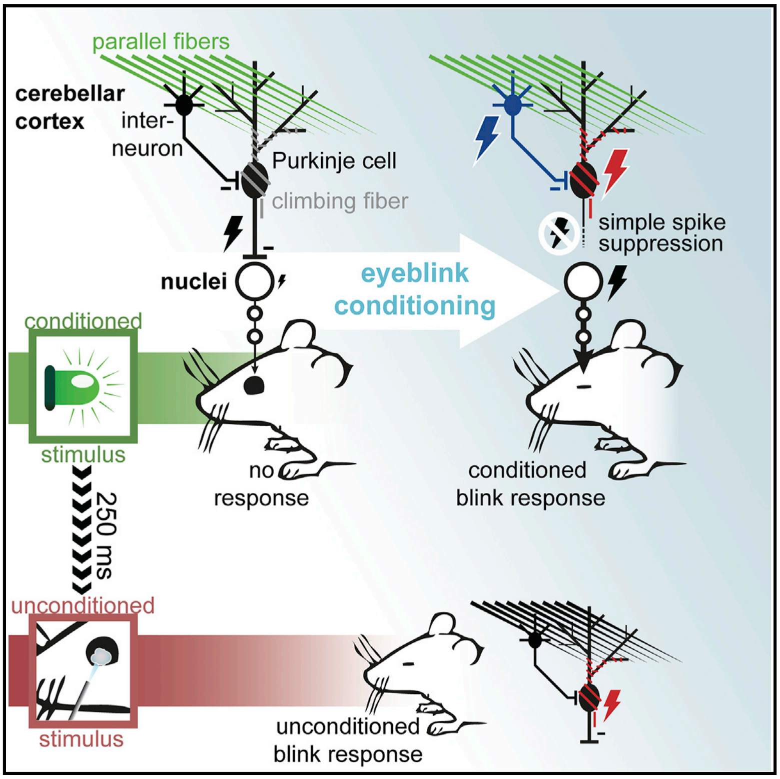

Highlights

- Simple spike suppression correlates trial by trial to conditioned eyelid behavior
- Conditioned stimulus-related complex spikes relate to simple spikes and behavior
- Molecular layer interneuron (MLI) modulation correlates to behavior
- Transgenic deficits in MLI input result in partially impaired eyeblink conditioning

Authors

Michiel M. ten Brinke, Henk-Jan Boele, Jochen K. Spanke, ..., Anna C.H.G. IJpelaar, Sebastiaan K.E. Koekkoek, Chris I. De Zeeuw

Correspondence

c.dezeeuw@erasmusmc.nl

In Brief

Purkinje cell simple spike suppression is a central driving mechanism in cerebellar conditioning. Here, ten Brinke et al. show how simple spike suppression, conditioned stimulus-related complex spikes, and molecular layer interneuron (MLI) activity correlate to conditioned eyelid behavior. Moreover, transgenic impairment of MLI input results in deficits in conditioned behavior.

ten Brinke et al., 2015, Cell Reports *13*, 1977–1988
December 1, 2015 ©2015 The Authors
http://dx.doi.org/10.1016/j.celrep.2015.10.057

Cell Reports
Article

OPEN ACCESS
CellPress

Evolving Models of Pavlovian Conditioning: Cerebellar Cortical Dynamics in Awake Behaving Mice

Michiel M. ten Brinke,[1,5] Henk-Jan Boele,[1,5] Jochen K. Spanke,[1,5] Jan-Willem Potters,[1] Katja Kornysheva,[1,2] Peer Wulff,[4] Anna C.H.G. IJpelaar,[1] Sebastiaan K.E. Koekkoek,[1] and Chris I. De Zeeuw[1,3,*]
[1]Department of Neuroscience, Erasmus Medical Center, 3000 Rotterdam, the Netherlands
[2]Institute of Cognitive Neuroscience, University College London, London WC1N 3AR, UK
[3]Netherlands Institute for Neuroscience, Royal Academy of Arts and Sciences (KNAW), 1105 Amsterdam, the Netherlands
[4]Physiologisches Institut, Christian-Albrechts-Universität, 24118 Kiel, Germany
[5]Co-first author
*Correspondence: c.dezeeuw@erasmusmc.nl
http://dx.doi.org/10.1016/j.celrep.2015.10.057
This is an open access article under the CC BY license (http://creativecommons.org/licenses/by/4.0/).

SUMMARY

Three decades of electrophysiological research on cerebellar cortical activity underlying Pavlovian conditioning have expanded our understanding of motor learning in the brain. Purkinje cell simple spike suppression is considered to be crucial in the expression of conditional blink responses (CRs). However, trial-by-trial quantification of this link in awake behaving animals is lacking, and current hypotheses regarding the underlying plasticity mechanisms have diverged from the classical parallel fiber one to the Purkinje cell synapse LTD hypothesis. Here, we establish that acquired simple spike suppression, acquired conditioned stimulus (CS)-related complex spike responses, and molecular layer interneuron (MLI) activity predict the expression of CRs on a trial-by-trial basis using awake behaving mice. Additionally, we show that two independent transgenic mouse mutants with impaired MLI function exhibit motor learning deficits. Our findings suggest multiple cerebellar cortical plasticity mechanisms underlying simple spike suppression, and they implicate the broader involvement of the olivocerebellar module within the interstimulus interval.

INTRODUCTION

The cerebellum offers the perfect neuronal territory within which to achieve an understanding of simple forms of learning and memory that flow “continuously from molecules and cells through synapses and circuits to behavior without any grossly embarrassing gaps in the middle” (Hansel et al., 2001). Pavlovian eyeblink conditioning illustrates this beautifully for an elementary building block in learning: the capacity to make associations (Gormezano et al., 1962). Imagine receiving an air puff on your eye a quarter second after seeing a green light-emitting diode (LED) turn on, over and over again. The air puff is an unconditional stimulus (US) in that it yields an unconditional blink response (UR). From the reflex loop underlying this blink, a signal is transmitted to the inferior olive and subsequently through climbing fibers to a subset of Purkinje cells in mainly the simplex lobule (hemispheric lobule VI [HVI]) of the cerebellar cortex (Figure 1A; Jirenhed et al., 2007; Mostofi et al., 2010). This climbing fiber signal causes a complex spike in these Purkinje cells, whose activity moderates cerebellar nuclear cells that ultimately innervate the same eyelid musculature targeted by the reflex loop. The same Purkinje cells also receive massive sensory input through the mossy fiber-parallel fiber system, in large part originating in the pontine nuclei (Figure 1A). Hidden within this sea of parallel fibers are signals encoding the green LED light, which by default is a neutral stimulus. Only on the condition that it is consistently paired with the air puff and only by virtue of the Purkinje cell's capacity to forge associations between parallel and climbing fiber signals will one learn to perform a well-timed, conditional blink response (CR) upon exposure to the light, which is thusly called the conditional stimulus (CS).

In tandem with lesion and stimulation research (Clark et al., 1984; Yeo et al., 1984, 1985a, 1985b; McCormick and Thompson, 1984a; Yeo and Hesslow, 1998), electrophysiology studies of increasing sophistication have extended and solidified our understanding of eyeblink conditioning across behavioral, circuitry, and cellular levels (McCormick and Thompson, 1984b; Berthier and Moore, 1986; Hesslow and Ivarsson, 1994; Green and Steinmetz, 2005; Kotani et al., 2006; Jirenhed et al., 2007; Halverson et al., 2015). It is now established using peripheral stimuli as well as direct stimulation of nuclei and fibers that Purkinje cells can acquire various types of simple spike modulation within classical conditioning paradigms (Jirenhed et al., 2007; Tracy and Steinmetz, 1998; Kotani et al., 2006). Importantly, Hesslow and colleagues have shown in decerebrate ferrets that Purkinje cells with reliable short-latency complex spike responses to the ipsilateral US quite uniformly acquire suppressive simple spike responses with properties that are very similar to behavioral CRs (Hesslow and Ivarsson, 1994; Jirenhed et al., 2007; Wetmore

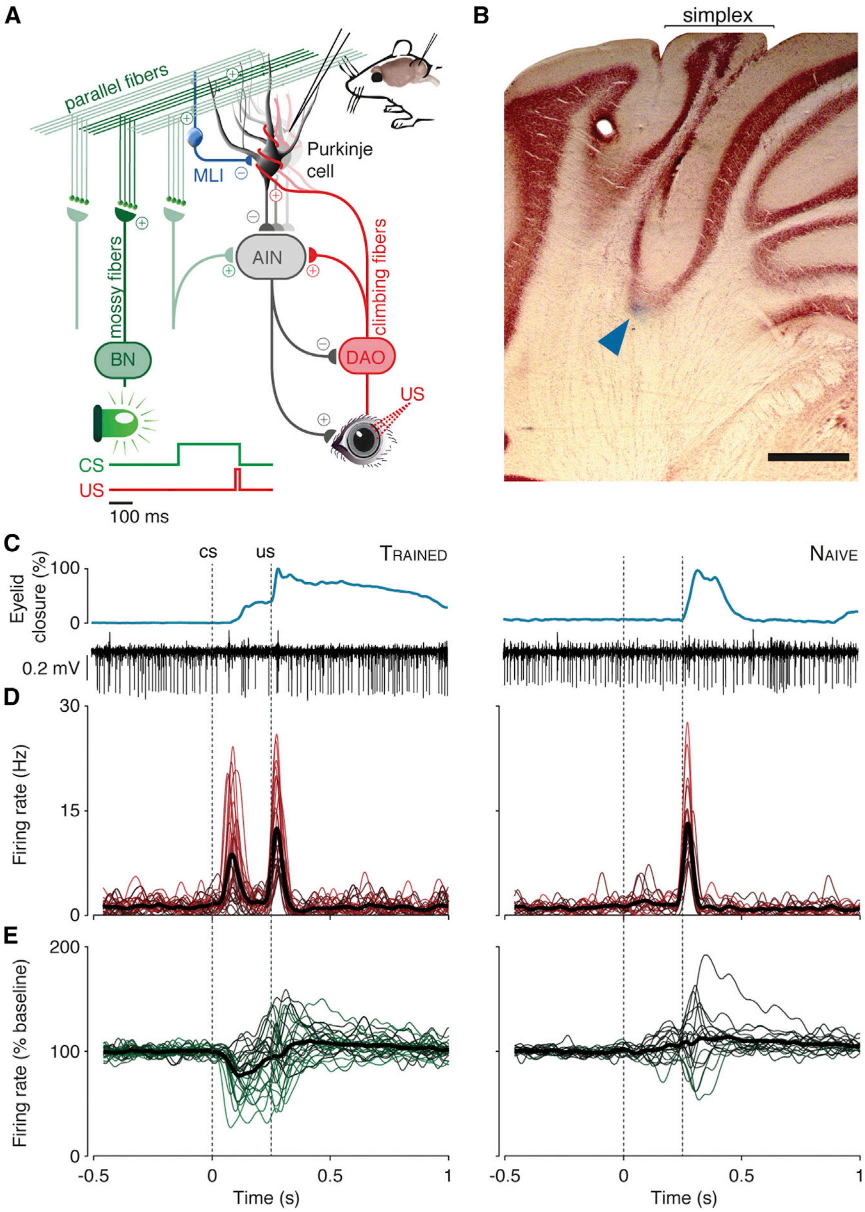

Figure 1. Purkinje Cell Characterization

(A) Purkinje cells in hemispheric lobule VI (zones C1/C3/D0) receive input from mossy fibers and climbing fibers, which carry CS and US signals, respectively. Simple spike suppression disinhibits the anterior interposed nuclei (AIN), which then drive CRs. Paired trials consisted of a 260-ms LED light CS co-terminating with a 10-ms corneal air puff.

(B) Coronal cerebellar section at −5.8 mm from bregma, with the blue arrow indicating staining of a typical extracellular recording site. The scale bar denotes 500 μm.

(C) Example eyelid and Purkinje cell traces for eyelid-related cells in trained animals (left column, n = 28) and in naive animals (right column, n = 17) are shown.

(D) SDFs of complex spikes for individual cells (strength of modulation relates to color brightness) and the mean for each set (thick black line) are shown.

(E) The same are shown for SDFs of simple spikes. BN, brainstem nuclei; DAO, dorsal accessory olive; MLI, molecular layer interneurons.

et al., 2014). Additionally, recent work in rabbits has begun drawing confirmative cross-correlations between the kinematic profile of eyelid behavior and simple spike activity in awake rabbits (Halverson et al., 2015).

The simple spike suppression observed in eyelid-related Purkinje cells seems in good agreement with the classical hypothesis dominating the synaptic level of cerebellar learning theory. Long-term depression (LTD) occurs at parallel fiber to Purkinje cell synapses when their activation is linked to climbing fiber activation (Ito et al., 1982; Coesmans et al., 2004; Gao et al., 2012). This plasticity mechanism is posited to constitute learning by suppressing simple spike firing in Purkinje cells, which in turn disinhibits the cerebellar nuclei, increasing cerebellar output (Marr, 1969; Albus, 1971; Ito, 2001). However, LTD seems neither necessary nor sufficient for eyeblink conditioning, as selective genetic or pharmacological blockage of parallel fiber to Purkinje cell LTD expression does not significantly impair eyeblink conditioning (Welsh et al., 2005; Schonewille et al., 2011), and short interstimulus intervals (ISIs) increase rather than decrease simple spike firing while presumably preserving LTD induction (Wetmore et al., 2008, 2014). In addition to Purkinje cells, parallel fiber activity encoding the CS reaches molecular layer interneurons (MLIs) through excitatory synapses, which can probably be strengthened through concomitant climbing fiber activity and, thereby, in principle, contribute to eyeblink conditioning (Gao et al., 2012). However, having shown that conditioned Purkinje cell simple spike suppression seems to persist after blocking cerebellar cortical feedforward synaptic inhibition provided by MLIs, Hesslow and colleagues are now homing in on potential mechanisms intrinsic to Purkinje cells (Johansson et al., 2014).

In the pursuit of a seamless understanding of Pavlovian conditioning across levels of analysis, we wish to address three considerable gaps that exist in current understanding. First, it remains to be seen how eyelid-related Purkinje cells in awake mice behave within a functional, undamaged brain, as existing work has relied heavily on the decerebrate preparation. Especially complex spike activity may depend to a large degree on circuitry level factors, which are likely different between awake and decerebrated preparations. Second, the potential disqualification of MLIs as a main mechanism underlying Purkinje cell simple

spike suppression (Johansson et al., 2014) raises the questions of what role they do carry, if and how they modulate in an eyeblink-conditioning paradigm, and to what extent their activity correlates with eyelid behavior. Finally, trial-by-trial quantification of simultaneously recorded conditioned eyelid behavior and cerebellar cortical activity in awake behaving animals is lacking so far. To bridge these gaps, we here present simultaneously recorded cerebellar cortical electrophysiology and eyelid behavior from awake behaving mice that were either naive or trained in an eyeblink-conditioning paradigm. Additionally, we present behavioral data obtained from two independent transgenic mouse mutants in which MLI inhibition was impaired through different mechanisms (Wulff et al., 2009; Seja et al., 2012).

RESULTS

Purkinje Cell Characterization

Our dataset comprises 57 Purkinje cells, 40 of which were recorded in trained mice and 17 in mice that were not previously trained. Guided by former studies (Heiney et al., 2014; Mostofi et al., 2010), we centered our recording area on the floor of the primary fissure (Figure 1B). We considered Purkinje cells to be eyelid related when they exhibited a complex spike response within 60 ms after the periocular air puff US in at least 20% of paired trials, correcting for chance occurrence. In the trained dataset, this criterion adequately separated 28 cells showing this US-complex spike response (henceforth, trained cells; Figures 1C–1E; Table 1) from 12 cells that did not (Figure S1). The untrained dataset consisted of only eyelid-related Purkinje cells (naive cells; Figures 1C and 1D) by virtue of meeting the US-complex spike criterion. Note that the latency of the consistent US-complex spike averaged 24.2 ms, but could range as far as 58 ms, which is why we used a 60-ms time range for the criterion. Although we refer to all conforming cells as eyelid related, the lack of a clear distinction of those showing short-latency US-complex spikes and those exhibiting longer ones, presumably due to our use of an air puff US instead of electrical stimulation, means our dataset could possibly also contain cells from zone C2, deemed non-essential for eyeblink behavior (Mostofi et al., 2010; Hesslow, 1994; Heiney et al., 2014).

Acquired Simple Spike Suppression and CS-Related Complex Spikes

In line with existing work (e.g., Kotani et al., 2006; Jirenhed et al., 2007), we found a uniquely strong prevalence of simple spike suppression in the trained cells (Figure 1E). In 14 trained cells, we found a clear prevalence of CS-related complex spike responses at a mean latency of 88.1 ms that occurred in at least 20% of trials (corrected for chance occurrence). One cell showed more than 20% complex spikes in the ISI, but at a much longer latency (189.7 ms; Table 1) and without the characteristically thin distribution found in the other cells (Figure 1D); hence, it was not considered a cell with a CS-complex spike response. In contrast, across the 17 naive cells, only one cell showed a CS-complex spike response, and this was in a mouse that actually started showing small CRs already during a second experiment. The uniquely high prevalences of simple spike suppression and CS-complex spikes in the trained group confirm that both phenomena developed over the course of conditioning.

Simple Spike Suppression Correlates with Conditioned Eyelid Behavior

Average significant simple spike suppression ranged from 7% to 42% across 20 trained cells (mean = 20.5%). Between cells, the average magnitude of suppression did not relate to the average CR amplitude, onset, or prevalence (each $p > 0.25$) observed during the recordings. However, on a trial-by-trial basis, the percentage simple spike suppression showed clear correlations with conditioned behavior. First, simple spike suppression was on average 22% higher in trials with a CR compared to those without, in a linear mixed-effects regression ($p < 0.0001$; Table S1A; see Experimental Procedures). Moreover, simple spike suppression correlated to CR amplitude on a trial-by-trial basis, as apparent from eight individually significant trained cells (Figure 2B; Table 1), as well as a similar mixed-effects model with random intercepts and slopes for each cell ($p < 0.0001$; Figure 2A; Table S1B; see Experimental Procedures), which estimated an increase in CR amplitude of 0.33 percentage points per unit increase of percentage simple spike suppression. This coefficient was 0.72 when just including the eight individually significant trained cells. Post hoc power analyses confirmed high statistical power for these and subsequent mixed models (Table S1).

The incorporation of individual slopes per cell in the mixed model was based on likelihood ratio tests and implies that certain cells showed suppression across a large range but predicted rather small differences in CR amplitude, whereas others only mildly suppressed but predicted a large range of CR amplitudes. Even considering the possible inclusion of non-essential (C2) Purkinje cells in the dataset, this variation was as apparent among just the most clearly correlating cells (Figure 2B). Together with the lack of correlation between the averages, this observation juxtaposes the clear correlation between simple spike suppression and CR amplitude with a probable dissociation between their average magnitudes.

Having determined significant correlation between simple spike firing and CR expression, we next explored its temporal distribution across the ISI with the following descriptive methodology. Trial-by-trial correlations of concomitant mean instantaneous simple spike firing frequency and eyelid position in 20-ms windows were made with 5-ms steps, resulting in the diagonal elements denoting R values in Figure 2D. Next we correlated simple spike activity with eyelid position at both earlier and later time points, resulting in the lower and upper triangular parts of a correlation matrix (Figure 2D; Figure S2). This matrix shows for each temporal configuration the average negative correlation of 11 trained cells that showed clear focal areas in the upper triangular part of the ISI range, i.e., negative correlations within the ISI between simple spike firing frequency and subsequent eyelid position (see Experimental Procedures; Figure S2).

Two observations stand out in this analysis. First, taking the net negative correlation within the ISI at each offset between spikes and behavior revealed that simple spike activity correlates most optimally to eyelid behavior 50 ms afterward. Second, there seem to be two focal areas of negative correlation shared

Table 1. Summary of Trained Purkinje Cell Recordings

Cell	Simple Spike Frequency (Hz)	Complex Spike Frequency (Hz)	CRs (%)	Mean CR Onset (ms)	Mean CR Amplitude (%)	Valid Trials	SSp Suppression in Last 200-ms ISI/Baseline (%)	SSp × CR Amplitude R Value	US-Complex Spikes (%, Corrected)	Mean US-Complex Spike Latency (ms)	CS-Complex Spikes in ISI (%, Corrected)	Mean CS-Complex Spike Latency (ms)
1	62.5	1.08	91.3	134	26.9	23	11.6[a]	0.086	23.9	33.0	12.1	–
2	97.6	1.24	92.3	99.6	29.1	26	−3.5	0.031	23.3	41.0	15.2	–
3	78.9	1.11	89.5	154.6	48.4	39	5.8[a]	0.250	22.3	31.0	14.3	–
4	78.5	1.76	94.4	115.0	44.4	18	−2.2	0.029	22.7	30.0	−16.3	–
5	162.8	1.79	68.2	170.2	31.9	22	−1.3	0.060	89.2	13.0	9.7	–
6	69.3	1.69	46.2	159.4	30.9	26	3.7	−0.345	74.5	10.0	23.1	102.3
7	89.4	1.06	45.7	182.1	35.2	35	7.1[a]	−0.172	36.5	28.0	5.0	–
8	61.9	1.98	28.6	169.1	44.4	49	1.9	0.309	20.8	41.0	−0.6	–
9	95.8	1.40	47.5	171.8	36.2	40	18.4[a]	−0.218	69.1	13.0	54.9	96.3
10	78.3	1.60	71.4	171.6	37.4	42	19.1[a]	0.240	52.3	15.0	50.5	90.1
11	108.2	1.28	43.4	172.7	34.6	76	42.4[a]	0.348[b]	42.3	14.0	45.6	103.8
12	114.2	1.22	97.4	101.8	51.5	39	5.0[b]	−0.303	26.0	16.0	18.3	–
13	107.7	1.39	100.0	121.3	50.8	51	31.1[a]	0.444[a]	32.8	14.0	39.7	94.0
14	84.0	1.02	100.0	117.2	52.3	33	31.4[a]	0.065	27.2	20.0	29.1	(189.7)
15	146.7	0.71	76.5	94.0	62.1	17	12.0[a]	0.146	25.2	24.0	5.9	–
16	127.7	0.43	71.4	129.3	60.2	14	21.3[a]	−0.123	54.6	12.0	32.1	74.7
17	115.7	1.55	65.2	110.6	48.9	23	26.5[a]	0.073	77.7	25.0	52.7	67.8
18	130.7	0.84	92.9	87.6	58.7	14	30.0[a]	0.113	52.1	23.0	57.6	73.7
19	113.6	1.52	50.0	97.6	29.3	16	18.7[a]	0.093	65.9	18.0	49.4	71.1
20	85.2	1.69	94.7	130.8	50.1	19	14.1[a]	0.624[a]	26.7	58.0	15.6	
21	104.2	1.41	40.0	157.3	26.9	50	8.9[a]	0.024	65.5	17.0	30.6	97.6
22	87.3	1.33	54.2	165.9	51.5	24	8.6[a]	−0.178	54.5	16.0	20.8	104.1
23	44.2	1.49	69.2	47.2	50.3	39	32.6[a]	0.626[a]	42.4	33.0	60.3	93.7
24	129.8	1.45	96.4	61.2	49.0	28	15.0[a]	0.415[b]	27.0	26.0	20.9	81.8
25	65.1	1.14	92.9	82.1	25.6	84	32.1[a]	0.451[a]	69.3	19.0	64.3	82.9
26	101.6	1.00	85.3	76.5	32.8	34	1.9	0.582[a]	20.6	20.0	−7.4	–
27	104.3	1.10	90.0	106.6	44.7	71	9.3[a]	0.410[a]	27.8	36.0	−0.1	–
28	62.3	1.50	43.9	113.3	22.9	23	3.0	−0.065	35.4	30.9	1.2	–
Mean	96.7	1.3	72.8	125	41.7	34.8	14.4	0.143	43.1	24.2	25.2	88.1

[a]$p < 0.01$.
[b]$p < 0.05$.

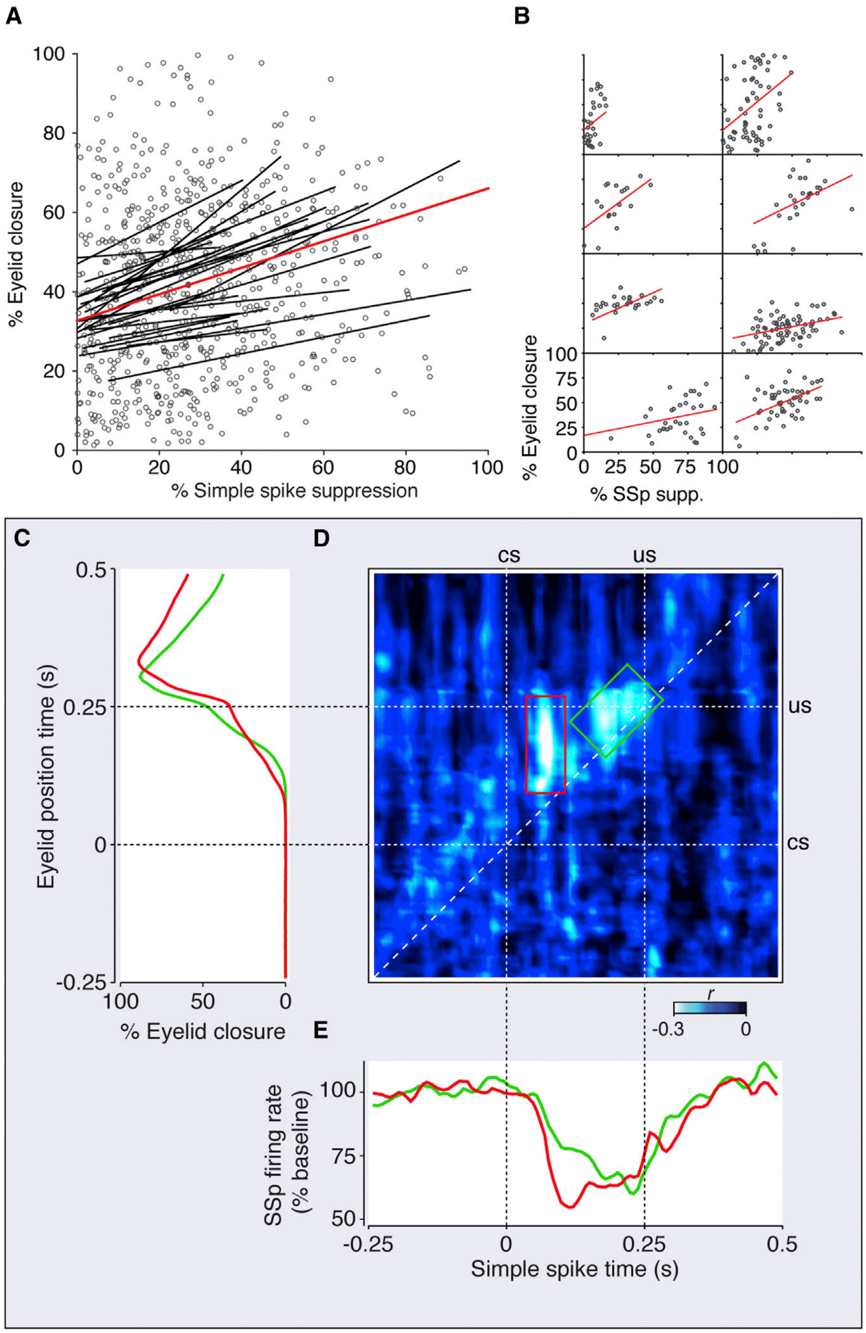

Figure 2. Simple Spike Suppression Relates to CR Expression

(A) Fit lines based on a linear mixed-effects regression, with random slopes and intercepts for each individual trained cell and the red line showing the full model fit. Each circle denotes a trial.

(B) Separated plots for eight trained cells that individually showed significant correlation between simple spike suppression and CR peak amplitude are given.

(C) Mean eyelid traces, rotated 90° to fit the y axis of the correlation matrix in (D). Red corresponds to seven trained cells showing correlation at the early focal area bound with a red box in (D), and green corresponds to four trained cells that correlated most in the later focal area.

(D) Averaged correlation matrix of 11 trained cells, detailing the temporal distribution of the negative correlation between simple spike activity and conditioned behavior. The two focal areas within the ISI indicated by the red and green boxes show the early and late spiking periods that correlate most prominently to subsequent eyelid behavior.

(E) SDFs of simple spikes for the same seven and four cells relating to the focal areas in the red and green boxes in (D), respectively, are shown.

between the 11 cells. Although the diagonal area at the end of the ISI is completely in line with expectations, the distinct focal area at the 100-ms mark is more surprising. It is positioned right around the time the CS-complex spike happens to occur, and its vertical orientation suggests a relatively short (~40-ms) window of reduced simple spike activity predicting the majority of the CR trajectory. The mean simple spike and eyelid traces for seven cells that showed these early focal areas (red) and four cells that did not (green) show latencies that are in compelling agreement with this distinction (Figures 2C and 2E). Although not significant cell-wise ($n = 11$, $p = 0.35$), the prevalence of CS-complex spikes was higher in the cells that showed early focal areas (median = 45.2%) than in those that did not (median = 22.4%).

CS-Complex Spikes Correlate with Simple Spike Suppression and Conditioned Eyelid Behavior

The occurrence of CS-complex spikes correlated to that of US-complex spikes between cells ($n = 28$, $r = 0.57$, $p = 0.0015$) and marginally to US-complex spike latency ($r = -0.384$, $p = 0.0437$). Among trained cells, mean percentage simple spike suppression was intimately connected to the prevalence of CS-complex spikes ($n = 28$, $r = 0.804$, $p < 0.0001$; Figure 3A). Additionally, a between-trial mixed-effects linear regression shows that suppression was on average 19.2% higher in trials with a CS-complex spike compared to those without ($p < 0.0001$; Table S1C). This translates to 9.2 ms of silence, a boost that could be sufficiently explained by the climbing fiber pause (mean = 16.5 ms).

As was the case with simple spike suppression, neither average CS-complex spike occurrence nor its average latency showed between-cell correlations to average CR properties (each $p > 0.25$), except for a small correlation between mean CS-complex spike latency and mean CR onset ($n = 14$, $r = 0.567$, $p = 0.0346$). However, on a trial-by-trial basis, mixed-effects regression on

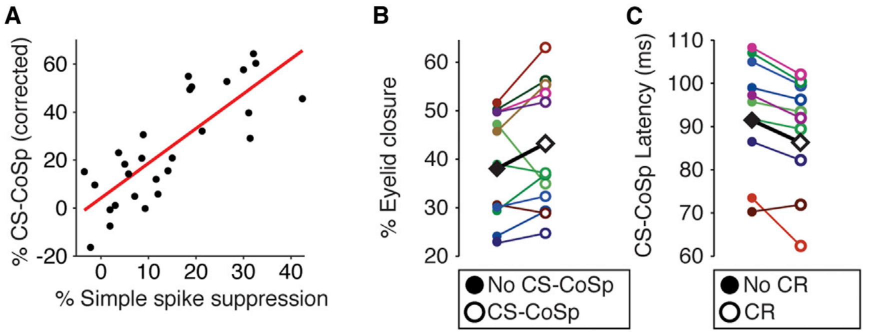

Figure 3. CS- and US-Related Complex Spikes Relate to CR Expression

(A) Between trained cells, the prevalence of CS-complex spikes correlated strongly to average percentage simple spike suppression ($n = 28$, $r = 0.803$, $p < 0.0001$).

(B) Trials with CS-complex spikes tended to exhibit higher CR amplitudes compared to those without. The black squares indicate the overall estimate from the mixed model ($p = 0.0194$).

(C) Conversely, latencies of CS-complex spikes for individual cells show that they tended to appear earlier in trials with a CR compared to those without ($p < 0.0001$).

the 14 cells with a clear CS-complex spike did show that CRs in trials with a CS-complex spike were on average 13.7% higher in amplitude than those without ($p = 0.0194$; Figure 3B; Table S1D). Also, in a similar mixed model, CR amplitude related inversely to CS-complex spike latency, with an average decrease in percentage eyelid closure of 0.26 percentage points per millisecond increase of CS-complex spike latency ($p = 0.0034$; Table S1E). Conversely, CS-complex spike latency was 4.9 ms earlier in trials with a CR compared to those without ($p < 0.0001$; Figure 3C; Table S1F). Together, these mild effects imply a modest contribution of CS-complex spikes to conditioned behavior on a trial-by-trial basis. The significance of the link between CS-complex spike occurrence and CR amplitude disappears when including simple spike suppression in the regression model ($p = 0.16$; Table S1G). This raises the possibility that CS-complex spikes affect conditioned behavior in part through their effect on simple spike suppression. Still, CS-complex spike latency retains some significance as a predictor of CR amplitude, even when including simple spikes in the model ($p = 0.0083$; Table S1H). This raises the possibility that climbing fiber signals and/or that of their collaterals in the nuclei directly contribute to the conditioned motor response.

MLI Activity Correlates with Conditioned Eyelid Behavior

Recent findings showed conditioned simple spike suppression in the absence of MLI feedforward inhibition by GABAergic neurotransmission (Johansson et al., 2014). Yet, the existence of conditioned CS-related complex spike responses reported here raises the possibility of conditioned CS-related MLI activation, either through glutamate spillover (Jörntell and Ekerot, 2003; Szapiro and Barbour, 2007) or ephaptic inhibition (Blot and Barbour, 2014). During our experiments, we recorded a set of 13 interneurons in the molecular layer (see Experimental Procedures; Ruigrok et al., 2011; Badura et al., 2013), all of which showed significant increases in their firing frequency in the ISI (Figures 4A and 4B; Table S2). Cell-wise, the mean onset of modulation correlated with the mean CR onset ($n = 13$, $r = 0.582$, $p = 0.03675$). Within the dataset, six MLIs individually showed significant positive trial-by-trial correlations between firing frequency in the ISI and CR amplitude; two cells showed significant negative correlations to CR amplitude (despite an overall increase in firing frequency in the ISI; Table S2). In one case, we were able to record a block of paired trials during the recording of an MLI immediately adjacent to a Purkinje cell from which we also recorded a block of paired trials. The latencies of the modulation of these cells were complementary (Figure 4C), as were their correlations to behavior (Figure 4D). Using the same correlation matrix approach explained above, the six positively correlating MLIs showed focal areas that complement those found in the Purkinje cell simple spike correlation matrix (Figure 2D), with a diagonal area focused near the end of the ISI and centered at an offset of approximately 50 ms between spikes and subsequent eyelid position, and again an early vertically oriented focal area. Here too, mean spike and eyelid traces for cells split on whether they showed this early focal area or not were in line with this distinction (Figure 4E). The two negatively correlating MLIs showed only a focal area near the end of the ISI (Figure 4F).

To investigate whether impairments in MLI function actually lead to any behavioral deficits, we subjected two independent cell-specific mouse lines, in which MLI function is impaired via completely different strategies, to behavioral eyeblink conditioning; these included the L7-Gamma2 mouse mutant, which lacks the Gamma2 subunit of the $GABA_A$ receptor in Purkinje cells (Wulff et al., 2009), and the L7-KCC2 mouse mutant, which lacks the potassium-chloride co-transporter KCC2 in Purkinje cells (Seja et al., 2012). In line with our expectations and despite potential developmental compensation, deficits in conditioned eyelid responses were found in both the L7-Gamma2 ($p = 0.0039$; Figure 4G) and L7-KCC2 ($p = 0.0029$; Figure 4H) mutants. Together with the fact that MLIs modulate in the ISI and even strongly correlate to behavior, these behavioral findings in two different knockout lines assert partial involvement of MLIs in the establishment of conditioned eyeblink behavior.

DISCUSSION

This work brings together electrophysiological data of Purkinje cells selected strictly based on the presence of consistent US-complex spike responses with precise behavioral recordings in an eyeblink conditioning paradigm employing awake behaving mice. In addition to strengthening the notion that the modulation observed in eyelid-related Purkinje cells is acquired and overwhelmingly suppressive, the current data expand on this finding by providing trial-by-trial quantification of the correlations between cellular spiking activity and eyelid behavior (Figures 5A and 5B). Across trials, Purkinje cell simple spike suppression correlates the strongest to conditioned eyelid behavior occurring

50 ms afterward. Importantly, we show the existence of a consistent CS-related complex spike response within the ISI, at an average of 88 ms after CS onset, that appears to be acquired and can be related to simple spike suppression and the behavioral CR. Finally, we provide evidence that there are MLIs in lobule simplex that increase their firing in the ISI and show mainly positive correlations to the amplitude of conditioned eyelid behavior, and that genetically impairing their inhibitory effect on Purkinje cells causes behavioral deficits in conditioning.

Conditioned Modulations of Purkinje Cell Activity and Eyelid Behavior

The current within-trial correlational data provide direct evidence for the intimate link between conditioned Purkinje cell simple spike suppression and behavioral CR expression. Taken together with the compelling similarities observed between the ways these neuronal and behavioral CRs are generally acquired, expressed, extinguished, and reacquired (Jirenhed et al., 2007; Wetmore et al., 2014; De Zeeuw and Ten Brinke, 2015) as well as the fact that suppression of Purkinje cell simple spikes through optogenetic stimulation of MLIs in lobule simplex can effectively elicit blink responses (Heiney et al., 2014), the inference of causality between Purkinje cell CRs and behavioral CRs is becoming increasingly unavoidable. We propose to expand this relationship to include the CS-related complex spike, a phenomenon mentioned as early as in Berthier and Moore (1986) (S. Edgley et al., 2010, Soc. Neurosci., abstract). Its virtual absence in our data from naive animals suggests that it is acquired, and its latency relative to the other cerebellar cortical components and conditioned eyelid behavior fits a potentially facilitating role, which is further supported by its significant correlations to both eyelid behavior and simple spike suppression (Figure 5).

This finding is important, because acquired climbing fiber activity within the ISI emphasizes the need to consider interplay within the broader network of olivocerebellar modules in the creation of conditioned motor responses. Indeed, the relatively mild, yet significant and positive, correlations found for trained cells on a trial-by-trial basis between CS-complex spike responses and conditioned behavior presumably present an underestimation of the actual contribution of CS-complex spikes to conditioned behavior, because the encoding of CS responses of ensembles of Purkinje cells within functional microzones usually surpasses that of individual Purkinje cells within such zones by far (Hoogland et al., 2015). The observed level of integration within the established model of cerebellar Pavlovian conditioning is also why we consider these CS-complex spike responses to be qualitatively different from the type reported in Rasmussen et al. (2014), who mention its presence in their training paradigm in decerebrate ferrets from the naive state on out, at predominantly 10- to 20-ms latencies rather than the 70- to 100-ms latencies that we observed in awake behaving mice.

Long US-Complex Spike Latencies Did Not Relate to Less Modulation or Correlation

Purkinje cells with long latency US-complex spikes should be regarded with caution in light of the findings by Mostofi et al. (2010), who showed in rabbits that these cells mainly originate in zone C2, which responds to more than just periocular stimulation and is unlikely to play an essential role in eyeblink conditioning (e.g., Yeo and Hesslow, 1998). In our recordings we were not able to find relevant differences based on US-complex spike latency with respect to the extent of simple spike suppression and its correlation to behavior or the occurrence of CS-complex spikes. Our air puff US may well be responsible for an exaggerated length and spread of latencies compared to electrical periocular stimulation, obfuscating a clear distinction between the two latency groups. The resultant possibility of the inclusion of C2 cells in our dataset warrants caution with inferences regarding effect size and spread. This is also true for cells that could respond to any non-eyelid-related parts of the US, improbable as it may be. However, given the compelling correlations found, it seems unlikely we here misattribute the reported positive links with eyeblink conditioning to actual eyeblink cells from zones C1/C3/D0 while they actually correspond to non-essential C2 cells, let alone to cells that are not at all eyelid related.

Temporal Dynamics Argue against a Single Mechanism Underlying Cerebellar Conditioning

The temporal dynamics observed between spike activity of cerebellar cortical neurons and eyelid behavior do not suggest a single focal area around which the cells' optimal correlations are dispersed. It is clear that the acquired simple spike suppression does not only gradually deepen to maximize around US onset time. In fact, our results hint at the possibility that there is also a particular process whose crucial window of action in the ISI is earlier on, centered around 80–100 ms after CS onset. The clear separation of these two focal areas in both the simple spike and MLI activity correlation matrix could imply differential contribution of plasticity mechanisms in cerebellar conditioning. This is certainly a plausible concept considering the variability observed in conditioned eyelid behavior in mice. Interestingly, the occurrence of CS-complex spikes strikingly overlaps with the early, but not the later, focal area in the correlation matrix, imparting this time window with remarkable significance. It is difficult to accommodate for this with a purely LTD-focused hypothesis or the notion of intrinsic plasticity at the level of single Purkinje cells. Why would an isolated time window early in the ISI show both a consistent complex spike response and particularly strong correlations between conditioned eyelid behavior and both simple spike and MLI activity?

The presence of the CS-complex spike means the CS-encoding signal has acquired the means to reach the olivary nucleus. Insofar as the eyeblink paradigm used here contains an operant component in that the CR can reduce the aversive impact of the air puff US (Longley and Yeo, 2014), cerebral cortical involvement might possibly be involved. In any case, the strong Purkinje cell-wise link between the occurrence of CS-complex spikes and the magnitude of simple spike suppression suggests interdependent plasticity mechanisms underlying both phenomena. An interesting possibility is that loops within the olivocerebellar network could play a role. The origin of the CS-complex spike could lie in the mossy fiber collaterals to the cerebellar nuclei that are newly formed over the course of conditioning (Boele et al., 2013), which could establish a straightforward bridge to

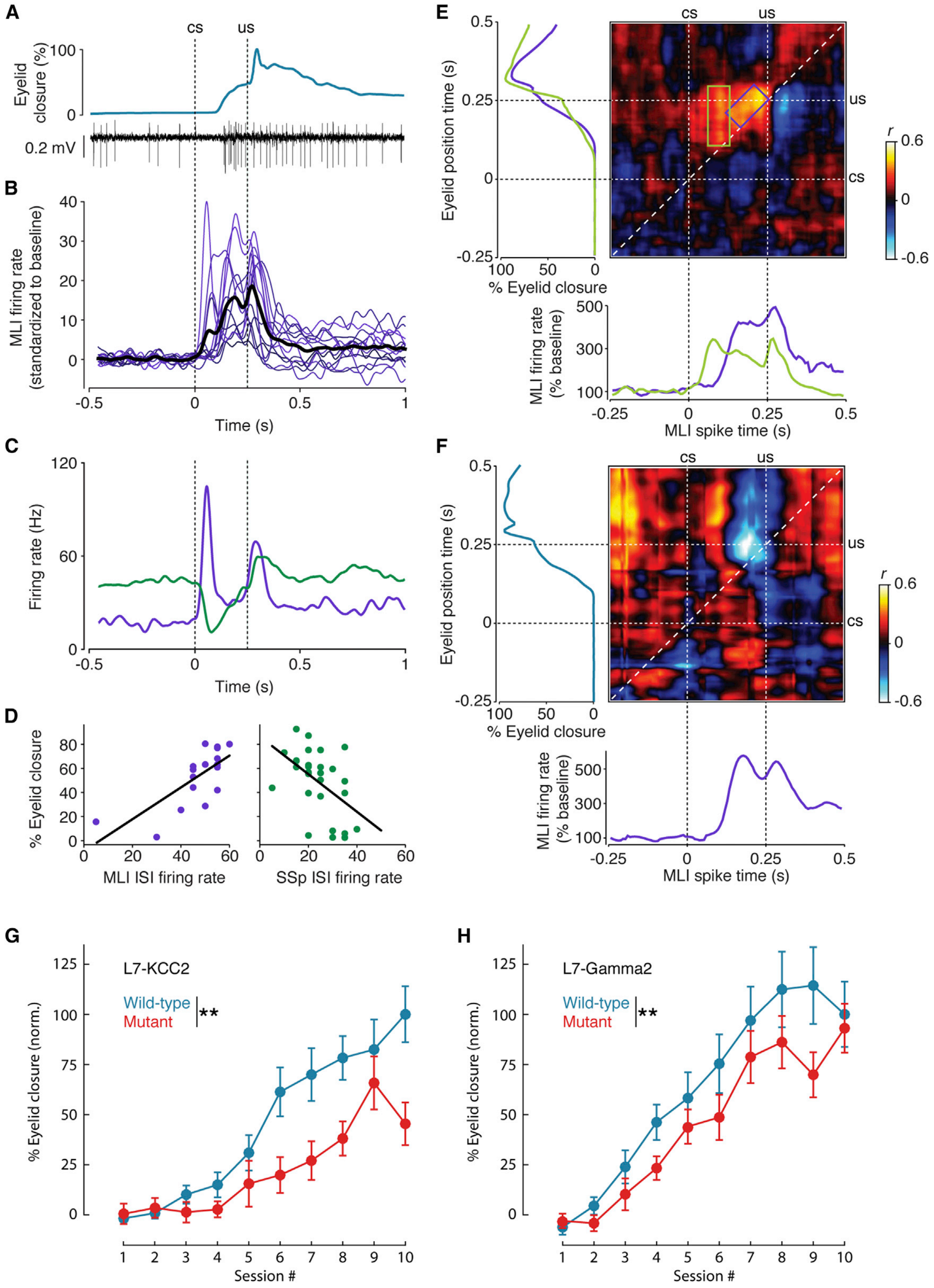

Figure 4. MLIs Modulate in the ISI and Correlate to CRs

(A) Example eyelid and electrophysiology traces during a paired trial while recording an MLI are shown.

(B) SDFs standardized to baseline for 13 MLIs (thick black line denotes mean trace) are shown.

(C) SDFs of a Purkinje cell recording (green) and a recording of an adjacent MLI (purple) encountered immediately afterward.

(legend continued on next page)

cross for CS-encoding signals to directly hook up to the nucleo-olivary pathway (De Zeeuw et al., 1988). The resulting CS-activated olivary inhibition could then bear rebound spikes (Bazzigaluppi et al., 2012; De Gruijl et al., 2012) that return to the cerebellum to affect cortical activity in a number of ways. It could influence simple spike suppression through the climbing fiber pause (De Zeeuw et al., 2011), through non-synaptic activation of MLIs (Jörntell and Ekerot, 2003; Szapiro and Barbour, 2007; Mathews et al., 2012), as well as through several climbing fiber-dependent forms of cerebellar cortical plasticity (Gao et al., 2012). Besides influencing simple spike suppression, it could play a role in higher-order conditioning, allowing the CS to be associated to novel input stimuli, preserving efficiently the same output pathway to the proper motor domain.

MLIs Partially Contribute to Conditioned Behavior

Our MLI data may seem at odds with the Johansson et al. (2014) study, because while the latter chemically dissociated MLI inhibition from the presence of simple spike suppression, we found strong modulation of interneuron spiking as well as correlations with behavioral CRs (Figures 4 and 5). However, these findings are not mutually exclusive. The possibility of a partial contribution of MLI activity to simple spike suppression and conditioned behavior seems probable from these different findings. Our behavioral data from two independent mouse mutants with impaired MLI inhibition showed partial deficits, which is much in line with this possibility, as is the feasibility of MLI-activated blinks generated by optogenetic stimulation (Heiney et al., 2014).

Conclusion

This study evinces the feasibility of simultaneous behavioral and neuronal recordings in awake behaving mice and strengthens the main tenet of cerebellar learning regarding suppressive modulation as a central process underlying learning (De Zeeuw et al., 2011; De Zeeuw and Ten Brinke, 2015). Although there is both theoretical and empirical support for interaction between the modulatory components of cerebellar cortical activity, they do not seem to share any strict interdependency from the perspective of individual Purkinje cells. After all, absence of CS-complex spikes alone does not necessarily prevent simple spike suppression altogether, nor does absence of feedforward inhibition through the MLIs (Johansson et al., 2014). Yet, the current findings in awake behaving mice suggest the involvement of CS-complex spikes and MLI activity in the conditioning paradigm is robust, further lending credence to the notion of distributed synergistic plasticity (Gao et al., 2012). Moreso, the CS-complex spike extends this notion beyond the cerebellar cortex to include recurrent activity within the whole olivocerebellar module at the level of the ISI. Of course, plasticity mechanisms intrinsic to Purkinje cells, such as those mediated by various mGluRs, phosphatases, and kinases may well be a decisive factor in eyeblink conditioning (Gao et al., 2012; Johansson et al., 2014). However, our results would at least stress caution regarding the inference of unitary localization of a plasticity process underlying conditioned simple spike suppression within only Purkinje cells. In the same vein, we also should point out that while LTD does not seem to be essential for eyeblink conditioning (Welsh et al., 2005; Schonewille et al., 2011; Wetmore et al., 2014), we cannot exclude the possibility that it still does contribute to the observed simple spike suppression, especially since the direction of its presumptive effect appears to be in line. However, the fact that impairments in potentiation mechanisms at the level of Purkinje cells cause behavioral deficits in eyeblink conditioning (Schonewille et al., 2010) underlines how the role of plasticity mechanisms may not play out as straightforwardly as one would expect. Our results show the importance of investigating network involvement in the acquisition and expression of Purkinje cell simple spike suppression. Specifically, unearthing the origin and function of CS-related complex spikes could further sophisticate cerebellar models of Pavlovian conditioning and advance our understanding of the strategies implemented by the brain to achieve learning.

EXPERIMENTAL PROCEDURES

Surgery

We used 12- to 20-week-old male wild-type C57Bl/6 (n = 34), L7-Gamma2 (mutants, n = 14; wild-type littermates, n = 11; see Wulff et al., 2009), and L7-KCC2 (mutants, n = 9; wild-type littermates, n = 11; see Seja et al., 2012) mice, housed individually with food and water ad libitum in a normal 12:12 light/dark cycle. The experiments were approved by the institutional animal welfare committee (Erasmus Medical Center). Mice were anesthetized with 2% isoflurane and body temperature was kept constant at 37°C. After fixation in a standard mouse stereotaxic alignment system (Stoelting), the scalp was opened to expose the skull. Membranous tissue was cleared and the bone was surgically prepared with Optibond prime and adhesive (Kerr). A small brass pedestal was attached to the skull with Charisma (Heraeus Kulzer), using an xyz manipulator, allowing for fixation to a head bar at right angles during training and electrophysiology. For the craniotomy performed after eyeblink acquisition training, skin and muscle tissue were cleared from the left half of the interparietal bone, where, after applying a local analgesic (bupivacaine hydrochloride 2.5 mg ml^{-1}), a roughly 1.5-mm-wide craniotomy centered at −5.7 mm from bregma and 2 mm from midline was performed, exposing the left cerebellar lobule simplex. A small rim of Charisma was made around the craniotomy and anti-inflammatory (Dexamethasone 4 mg ml^{-1}) solution was applied inside, after which the chamber was closed with a very low viscosity silicone elastomer sealant (Kwik-cast, World Precision Instruments).

(D) The activities of the MLI and Purkinje cell in (C) show opposite significant correlations to CR peak amplitude (n = 17, r = 0.734, p = 0.0007; n = 28, r = −0.498, p = 0.0071, respectively).

(E) Correlation matrix derived from combined standardized data of six MLIs showing significant positive correlations within the ISI, distributed across two focal areas similar to the matrix for simple spikes (Figure 2D). Mean eyelid traces (left panel) and spike traces (bottom panel) corresponding to four cells that showed the early focal area (green box) and two that did not (purple box) are shown.

(F) Correlation matrix shows the temporal distribution of the negative correlations found in two interneurons, similarly accompanied by mean eyelid spike trace.

(G) Learning curves for L7-KCC2 mutants (red, n = 9) and wild-type littermates (blue, n = 13) were computed by taking the average percentage eyelid closure right at US onset time for all trials (100 per session). Error bars denote SEM.

(H) Learning curves, similarly computed, for L7-Gamma2 mutants (red, n = 14) and wild-type littermates (blue, n = 11). Percentages of eyelid closures in (G) and (H) were normalized for comparison across the mutant groups, which were derived from different backgrounds (Wulff et al., 2009; Seja et al., 2012).

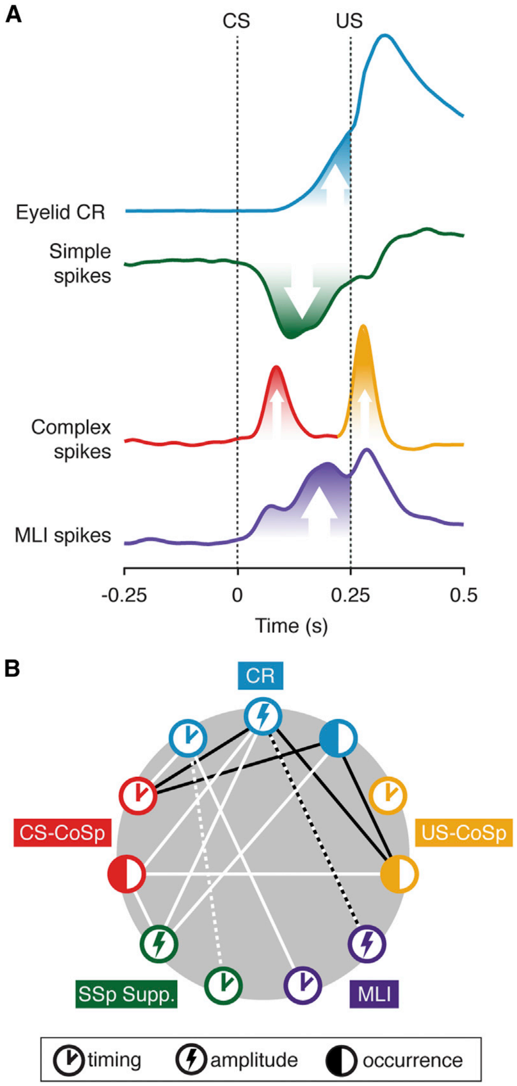

Figure 5. Cerebellar Cortical Activity and Conditioned Eyelid Behavior: Modulation and Correlation

(A) Mean eyelid, simple spike, and complex spike traces of all cells in the trained dataset and mean MLI spike trace of all cells in the MLI dataset show how the modulatory components in the cerebellar cortex relate after training.

(B) Diagram depicting for each of the components in (A) the significant correlations existing between their different properties (timing, amplitude, and occurrence). White lines denote positive correlations, black lines denote negative ones. Note that the lack of connections between MLI and PC-related nodes reflects the fact that we did not record simultaneously from these types of neurons, rather than a lack of correlation between their properties. The white line connecting simple spike timing to CR timing is dashed because we could not reliably quantify onset of simple spike suppression on a per-trial basis to enable robust statistical testing. We would have been remiss not to make some connection, since the correlation matrix does show a clear temporal link between the two components.

Training

Two days after surgery, mice were head-fixed to a brass bar suspended over a cylindrical treadmill (Chettih et al., 2011) and were placed in a sound- and light-isolating chamber for their first habituation session, which consisted of 30 min during which no stimuli were presented. During second and third habituation sessions on consecutive days, ten CS-only trials were presented to acquire a baseline measurement. The next 10 days mice received 100 paired trials daily, with an inter-trial interval of 10 ± 2 s, amounting to sessions that lasted approximately half an hour each.

The CS was a 260-ms green LED light placed ~7 cm in front of the mouse. The US was a 10-ms corneal air puff at 40 psi delivered through a 27.5G needle tip positioned ~5 mm from the left eye, co-terminating with the CS, which amounted to an ISI of 250 ms. TDT System 3 (Tucker Davis Technologies) and NI-PXI (National Instruments) processors were used to trigger and keep track of stimuli while capturing data. Eyelid movements were recorded with either the magnetic distance measurement technique (MDMT) at 1,017.26 Hz or a 250-frame/s camera (scA640-120 gc, Basler). Both methods are explained and shown to reflect eyelid movements with high accuracy (Koekkoek et al., 2002).

Electrophysiology

Before proceeding with the neuronal recordings, mice were allowed several days to get used to the electrophysiology setup, which was located in a dedicated room and contained a similar eyeblink apparatus within a light-isolated Faraday cage. During this period, trained mice received daily training sessions consisting of 100 trials for a maximum of 4 days to ensure at least 75% CRs. Neurons were recorded with glass capillaries (Ø = 2 mm, Harvard Apparatus) that were heated and pulled to obtain a 2- to 5-μm tip and filled with a 2M NaCl solution. The electrode was lowered into lobule simplex (Heiney et al., 2014; Van Der Giessen et al., 2008) using a one-axis hydraulic manipulator (MMO-220A, Narishige). The obtained electrical signal was pre-amplified and digitized at a sampling frequency of 25 kHz using a TDT System 3 electrophysiology workstation. When a recording was stable, the animal was subjected to blocks of paired trials. Purkinje cells were identified by the presence of complex spikes. When we encountered cells that were located no further than 100 μm from Purkinje cells, had relatively low firing rates, and did not show complex spikes, we would record them if they seemed to modulate within the ISI. Offline analysis was used to verify these putative MLIs (see below).

Eyeblink Data Analysis

For each recording, eyelid traces were normalized to the full blink range, which consisted of the minimal resting baseline value reflecting the open eye position as established visually during the experiment and the mean of the unconditioned response peak values reflecting the closed eye position. The traces were smoothed using a second degree Savitzky-Golay method. An iterative Grubbs' outlier detection test ($\alpha = 0.05$) on trial baseline SDs was used to remove trials that had an unstable baseline. Next trials were considered to contain a CR when eyelid closure exceeded 10 SDs from the baseline mean within the ISI. CR amplitude was quantified as the maximum eyelid position within the ISI relative to the trial baseline position, expressed as a percentage of full blink range. CR onset was determined as the first time point of a continuous positive eyelid velocity leading to up to the fifth percentile of the amplitude from baseline to CR peak.

Spike Analysis

Electrophysiological recordings were analyzed in MATLAB (MathWorks) using custom-written code and SpikeTrain (Neurasmus). Extracellular waveforms were band-pass filtered at 150–6,000 Hz. The spike threshold was set at three SDs below mean signal by default, and for Purkinje cells an additional threshold at 3 SDs above mean signal ensured that no complex spikes were missed. After thresholding, spike shapes were analyzed, with particular attention paid to the negative spike amplitude, spike width, amplitude of the positive inflection immediately after the spike, frequency components after the spike, and possible spikelet occurrence at specific time points. Complex and simple spike clusters were identifiable for all Purkinje cells by plotting combinations of these spike properties against each other. Manual selections of ~75 simple spikes and as many complex spikes served as training sets for multilinear discriminant analysis (MLDA), which was used to label all thresholded spikes as either a simple or complex spike on the basis of the spike properties. This approach provides an adequate sort while keeping the clustering procedure tied to real and intuitive variables. Clear 8- to 15-ms climbing fiber pauses confirmed that the sorted simple and complex spikes belonged to a single

Purkinje cell. Spike density functions (SDFs) were computed for all trials by convolving simple and complex spike occurrences across 1-ms bins with a 41-ms Gaussian kernel (Figure S2).

After ascertaining whether a cell showed a clear CS-complex spike response (>20% of trials), we considered individual complex spikes to be CS-related if they occurred within the time range in which the average complex spike SDF was more than 2.5 SDs over baseline activity. For correlational analyses involving simple spikes, the SDFs were normalized so the mean of the 500-ms baseline was 1. The first 50 ms of the ISI was excluded, as neuronal modulation was not expected to occur there (e.g., Jirenhed et al., 2007). For the last 200 ms, simple spike modulation was quantified in individual trials as the total downward or upward deviation from mean baseline, divided by the expected (mean baseline) activity in the same time range. Specifically, the 200 data points (each 1 ms) were split into the values under mean baseline (SDF_{under}) and those that were over (SDF_{over}), with the following calculation resulting in the percentage of suppression and facilitation, respectively:

$$0.5 * \sum 1 - SDF_{under(i)} \quad \text{and} \quad 0.5 * \left(\sum SDF_{over(i)} - 1\right),$$

where i indexes the 200 ISI data points of one trial. Whether a cell modulated significantly was determined by comparing its suppression/facilitation percentages in the ISI to the deviation from baseline that occurred within the baseline itself, which was similarly computed. The non-Purkinje neurons recorded during experiments were identified as MLIs using the same process as described in Badura et al. (2013); we only appraised cells as MLIs if they passed a decision tree that incorporates various firing properties (e.g., Hz, CV, CV2, and interspike interval) and is outlined in Ruigrok et al. (2011). For MLIs, the difference between ISI firing rate and baseline firing rate was used to reflect modulation.

Statistics

Significance tests were performed using MATLAB. Pearson correlations were used in all single-cell analyses involving two continuous variables, after both variables were subjected to Grubbs' test for outliers ($\alpha = 0.05$). Single-cell analyses involving one dichotomous and one continuous variable (also tested for outliers) were Wilcoxon rank-sum tests in all correlations, except for the ones quantifying modulation in the ISI relative to baseline and the difference in CR amplitude between MLI mutants and wild-types across sessions, which was tested with a Wilcoxon matched-pair signed-rank test. For combined analysis of multiple cells, mixed-effects linear regression was performed with cells and animals as random effects.

Visual inspection revealed no obvious deviations from normality and homoscedasticity and helped identify extreme outliers (never more than four). Likelihood ratio tests were used to determine the appropriate random effects structure, which ended up never to include animal as a random effect. Statistical power of the mixed models was calculated using post hoc Monte Carlo simulations. This method takes into account the distribution of the random effects and that of the residuals present in the model, and it generates simulated output data based on random sampling from these distributions, using the same sample sizes as in the original model. By reiterating this process 500 times, recording for each instance the p values of the fixed effects (that were significant in the original model). The ratio of significant p values indicates the probability of detecting the effect upon replication using a similar model design and similar sample sizes and averaged 91.9% across the mixed models presented here (Table S1).

SUPPLEMENTAL INFORMATION

Supplemental Information includes two figures and two tables and can be found with this article online at http://dx.doi.org/10.1016/j.celrep.2015.10.057.

AUTHOR CONTRIBUTIONS

Experimental Design, M.M.t.B., H.-J.B., S.K.E.K., and C.I.D.Z.; Experiments, M.M.t.B., J.K.S., J.-W.P., H.-J.B., P.W., and A.C.H.G.I.; Data Analysis, M.M.t.B., J.K.S., K.K., H.-J.B., and C.I.D.Z.; Manuscript, M.M.t.B., C.I.D.Z., H.-J.B., K.K., and P.W. All authors discussed the results and commented on the manuscript. M.M.t.B., H.-J.B., and J.K.S. contributed equally to this study.

ACKNOWLEDGMENTS

The authors are grateful to Dimitris Rizopoulos for statistical guidance and Joost van Dongen, Patty Bacilio, Mariska Nagtzaam, Erika Goedknegt, and Elise Haasdijk for technical assistance. We thank the Dutch Organization for Medical Sciences (ZonMw Top-Go 912.100.00; C.I.D.Z.), Life Sciences (ALW 854.10.004; C.I.D.Z.), Senter (Neurobsik FES 0908 min VWS; C.I.D.Z.), the ERC-advanced (294775), Cerebnet (238686 ITN), and C7 (238214 ITN) programs of the European Community (C.I.D.Z.) and The Wellcome Trust (098881/Z/12/Z; K.K.) for their financial support.

Received: March 25, 2015
Revised: July 8, 2015
Accepted: October 16, 2015
Published: November 19, 2015

REFERENCES

Albus, J.S. (1971). A theory of cerebellar function. Math. Biosci. *10*, 25–61.

Badura, A., Schonewille, M., Voges, K., Galliano, E., Renier, N., Gao, Z., Witter, L., Hoebeek, F.E., Chédotal, A., and De Zeeuw, C.I. (2013). Climbing fiber input shapes reciprocity of Purkinje cell firing. Neuron *78*, 700–713.

Bazzigaluppi, P., Ruigrok, T., Saisan, P., De Zeeuw, C.I., and de Jeu, M. (2012). Properties of the nucleo-olivary pathway: an in vivo whole-cell patch clamp study. PLoS ONE *7*, e46360.

Berthier, N.E., and Moore, J.W. (1986). Cerebellar Purkinje cell activity related to the classically conditioned nictitating membrane response. Exp. Brain Res. *63*, 341–350.

Blot, A., and Barbour, B. (2014). Ultra-rapid axon-axon ephaptic inhibition of cerebellar Purkinje cells by the pinceau. Nat. Neurosci. *17*, 289–295.

Boele, H.J., Koekkoek, S.K., De Zeeuw, C.I., and Ruigrok, T.J. (2013). Axonal sprouting and formation of terminals in the adult cerebellum during associative motor learning. J. Neurosci. *33*, 17897–17907.

Chettih, S.N., McDougle, S.D., Ruffolo, L.I., and Medina, J.F. (2011). Adaptive timing of motor output in the mouse: the role of movement oscillations in eyelid conditioning. Front. Integr. Nueurosci. *5*, 72.

Clark, G.A., McCormick, D.A., Lavond, D.G., and Thompson, R.F. (1984). Effects of lesions of cerebellar nuclei on conditioned behavioral and hippocampal neuronal responses. Brain Res. *291*, 125–136.

Coesmans, M., Weber, J.T., De Zeeuw, C.I., and Hansel, C. (2004). Bidirectional parallel fiber plasticity in the cerebellum under climbing fiber control. Neuron *44*, 691–700.

De Gruijl, J.R., Bazzigaluppi, P., de Jeu, M.T., and De Zeeuw, C.I. (2012). Climbing fiber burst size and olivary sub-threshold oscillations in a network setting. PLoS Comput. Biol. *8*, e1002814.

De Zeeuw, C.I., and Ten Brinke, M.M. (2015). Motor Learning and the Cerebellum. Cold Spring Harb. Perspect. Biol. *7*, a021683. http://dx.doi.org/10.1101/cshperspect.a021683.

De Zeeuw, C.I., Holstege, J.C., Calkoen, F., Ruigrok, T.J., and Voogd, J. (1988). A new combination of WGA-HRP anterograde tracing and GABA immunocytochemistry applied to afferents of the cat inferior olive at the ultrastructural level. Brain Res. *447*, 369–375.

De Zeeuw, C.I., Hoebeek, F.E., Bosman, L.W., Schonewille, M., Witter, L., and Koekkoek, S.K. (2011). Spatiotemporal firing patterns in the cerebellum. Nat. Rev. Neurosci. *12*, 327–344.

Gao, Z., van Beugen, B.J., and De Zeeuw, C.I. (2012). Distributed synergistic plasticity and cerebellar learning. Nat. Rev. Neurosci. *13*, 619–635.

Gormezano, I., Schneiderman, N., Deaux, E., and Fuentes, I. (1962). Nictitating membrane: classical conditioning and extinction in the albino rabbit. Science *138*, 33–34.

Green, J.T., and Steinmetz, J.E. (2005). Purkinje cell activity in the cerebellar anterior lobe after rabbit eyeblink conditioning. Learn. Mem. *12*, 260–269.

Halverson, H.E., Khilkevich, A., and Mauk, M.D. (2015). Relating cerebellar purkinje cell activity to the timing and amplitude of conditioned eyelid responses. J. Neurosci. *35*, 7813–7832.

Hansel, C., Linden, D.J., and D'Angelo, E. (2001). Beyond parallel fiber LTD: the diversity of synaptic and non-synaptic plasticity in the cerebellum. Nat. Neurosci. *4*, 467–475.

Heiney, S.A., Kim, J., Augustine, G.J., and Medina, J.F. (2014). Precise control of movement kinematics by optogenetic inhibition of Purkinje cell activity. J. Neurosci. *34*, 2321–2330.

Hesslow, G. (1994). Correspondence between climbing fibre input and motor output in eyeblink-related areas in cat cerebellar cortex. J. Physiol. *476*, 229–244.

Hesslow, G., and Ivarsson, M. (1994). Suppression of cerebellar Purkinje cells during conditioned responses in ferrets. Neuroreport *5*, 649–652.

Hoogland, T.M., De Gruijl, J.R., Witter, L., Canto, C.B., and De Zeeuw, C.I. (2015). Role of Synchronous Activation of Cerebellar Purkinje Cell Ensembles in Multi-joint Movement Control. Curr. Biol. *25*, 1157–1165.

Ito, M. (2001). Cerebellar long-term depression: characterization, signal transduction, and functional roles. Physiol. Rev. *81*, 1143–1195.

Ito, M., Sakurai, M., and Tongroach, P. (1982). Climbing fibre induced depression of both mossy fibre responsiveness and glutamate sensitivity of cerebellar Purkinje cells. J. Physiol. *324*, 113–134.

Jirenhed, D.A., Bengtsson, F., and Hesslow, G. (2007). Acquisition, extinction, and reacquisition of a cerebellar cortical memory trace. J. Neurosci. *27*, 2493–2502.

Johansson, F., Jirenhed, D.A., Rasmussen, A., Zucca, R., and Hesslow, G. (2014). Memory trace and timing mechanism localized to cerebellar Purkinje cells. Proc. Natl. Acad. Sci. USA *111*, 14930–14934.

Jörntell, H., and Ekerot, C.F. (2003). Receptive field plasticity profoundly alters the cutaneous parallel fiber synaptic input to cerebellar interneurons in vivo. J. Neurosci. *23*, 9620–9631.

Koekkoek, S.K., Den Ouden, W.L., Perry, G., Highstein, S.M., and De Zeeuw, C.I. (2002). Monitoring kinetic and frequency-domain properties of eyelid responses in mice with magnetic distance measurement technique. J. Neurophysiol. *88*, 2124–2133.

Kotani, S., Kawahara, S., and Kirino, Y. (2006). Purkinje cell activity during classical eyeblink conditioning in decerebrate guinea pigs. Brain Res. *1068*, 70–81.

Longley, M., and Yeo, C.H. (2014). Distribution of neural plasticity in cerebellum-dependent motor learning. Prog. Brain Res. *210*, 79–101.

Marr, D. (1969). A theory of cerebellar cortex. J. Physiol. *202*, 437–470.

Mathews, P.J., Lee, K.H., Peng, Z., Houser, C.R., and Otis, T.S. (2012). Effects of climbing fiber driven inhibition on Purkinje neuron spiking. J. Neurosci. *32*, 17988–17997.

McCormick, D.A., and Thompson, R.F. (1984a). Cerebellum: essential involvement in the classically conditioned eyelid response. Science *223*, 296–299.

McCormick, D.A., and Thompson, R.F. (1984b). Neuronal responses of the rabbit cerebellum during acquisition and performance of a classically conditioned nictitating membrane-eyelid response. J. Neurosci. *4*, 2811–2822.

Mostofi, A., Holtzman, T., Grout, A.S., Yeo, C.H., and Edgley, S.A. (2010). Electrophysiological localization of eyeblink-related microzones in rabbit cerebellar cortex. J. Neurosci. *30*, 8920–8934.

Rasmussen, A., Jirenhed, D.A., Wetmore, D.Z., and Hesslow, G. (2014). Changes in complex spike activity during classical conditioning. Front. Neural Circuits *8*, 90.

Ruigrok, T.J., Hensbroek, R.A., and Simpson, J.I. (2011). Spontaneous activity signatures of morphologically identified interneurons in the vestibulocerebellum. J. Neurosci. *31*, 712–724.

Schonewille, M., Belmeguenai, A., Koekkoek, S.K., Houtman, S.H., Boele, H.J., van Beugen, B.J., Gao, Z., Badura, A., Ohtsuki, G., Amerika, W.E., et al. (2010). Purkinje cell-specific knockout of the protein phosphatase PP2B impairs potentiation and cerebellar motor learning. Neuron *67*, 618–628.

Schonewille, M., Gao, Z., Boele, H.J., Veloz, M.F., Amerika, W.E., Simek, A.A., De Jeu, M.T., Steinberg, J.P., Takamiya, K., Hoebeek, F.E., et al. (2011). Reevaluating the role of LTD in cerebellar motor learning. Neuron *70*, 43–50.

Seja, P., Schonewille, M., Spitzmaul, G., Badura, A., Klein, I., Rudhard, Y., Wisden, W., Hübner, C.A., De Zeeuw, C.I., and Jentsch, T.J. (2012). Raising cytosolic Cl- in cerebellar granule cells affects their excitability and vestibulo-ocular learning. EMBO J. *31*, 1217–1230.

Szapiro, G., and Barbour, B. (2007). Multiple climbing fibers signal to molecular layer interneurons exclusively via glutamate spillover. Nat. Neurosci. *10*, 735–742.

Tracy, J.A., and Steinmetz, J.E. (1998). Purkinje cell responses to pontine stimulation CS during rabbit eyeblink conditioning. Physiol. Behav. *65*, 381–386.

Van Der Giessen, R.S., Koekkoek, S.K., van Dorp, S., De Gruijl, J.R., Cupido, A., Khosrovani, S., Dortland, B., Wellershaus, K., Degen, J., Deuchars, J., et al. (2008). Role of olivary electrical coupling in cerebellar motor learning. Neuron *58*, 599–612.

Welsh, J.P., Yamaguchi, H., Zeng, X.H., Kojo, M., Nakada, Y., Takagi, A., Sugimori, M., and Llinás, R.R. (2005). Normal motor learning during pharmacological prevention of Purkinje cell long-term depression. Proc. Natl. Acad. Sci. USA *102*, 17166–17171.

Wetmore, D.Z., Mukamel, E.A., and Schnitzer, M.J. (2008). Lock-and-key mechanisms of cerebellar memory recall based on rebound currents. J. Neurophysiol. *100*, 2328–2347.

Wetmore, D.Z., Jirenhed, D.A., Rasmussen, A., Johansson, F., Schnitzer, M.J., and Hesslow, G. (2014). Bidirectional plasticity of Purkinje cells matches temporal features of learning. J. Neurosci. *34*, 1731–1737.

Wulff, P., Schonewille, M., Renzi, M., Viltono, L., Sassoè-Pognetto, M., Badura, A., Gao, Z., Hoebeek, F.E., van Dorp, S., Wisden, W., et al. (2009). Synaptic inhibition of Purkinje cells mediates consolidation of vestibulo-cerebellar motor learning. Nat. Neurosci. *12*, 1042–1049.

Yeo, C.H., and Hesslow, G. (1998). Cerebellum and conditioned reflexes. Trends Cogn. Sci. *2*, 322–330.

Yeo, C.H., Hardiman, M.J., and Glickstein, M. (1984). Discrete lesions of the cerebellar cortex abolish the classically conditioned nictitating membrane response of the rabbit. Behav. Brain Res. *13*, 261–266.

Yeo, C.H., Hardiman, M.J., and Glickstein, M. (1985a). Classical conditioning of the nictitating membrane response of the rabbit. I. Lesions of the cerebellar nuclei. Exp. Brain Res. *60*, 87–98.

Yeo, C.H., Hardiman, M.J., and Glickstein, M. (1985b). Classical conditioning of the nictitating membrane response of the rabbit. II. Lesions of the cerebellar cortex. Exp. Brain Res. *60*, 99–113.

Cell Reports

Supplemental Information

Evolving Models of Pavlovian Conditioning: Cerebellar Cortical Dynamics in Awake Behaving Mice

Michiel M. ten Brinke, Henk-Jan Boele, Jochen K. Spanke, Jan-Willem Potters, Katja Kornysheva, Peer Wulff, Anna C.H.G. IJpelaar, Sebastiaan K.E. Koekkoek, and Chris I. De Zeeuw

Figure S1

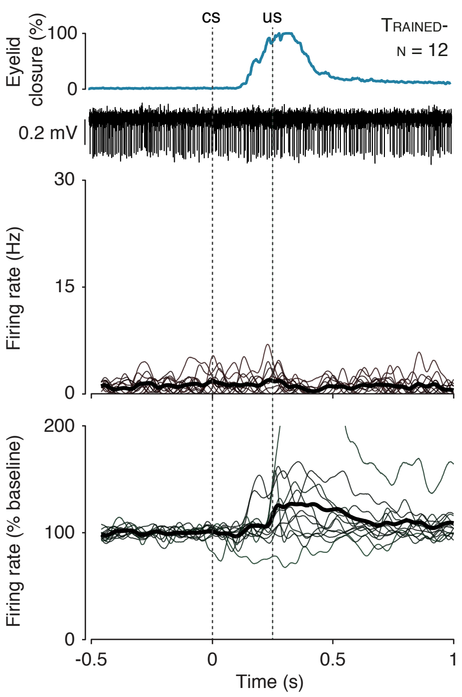

Figure S1. Purkinje cells without US-related complex spikes, Related to Figure 1. Example behavioral and spike trace in the top panel, complex spike density functions for twelve Purkinje cells in the middle panel, simple spike density functions in the bottom panel. These cells did not show US-complex spike responses and were recorded in trained mice. Seven cells showed no significant simple spike modulation, one showed suppressive modulation, and three showed facilitation in the ISI. This facilitation was not observed in cells with a clear complex spike response to the US.

Figure S2

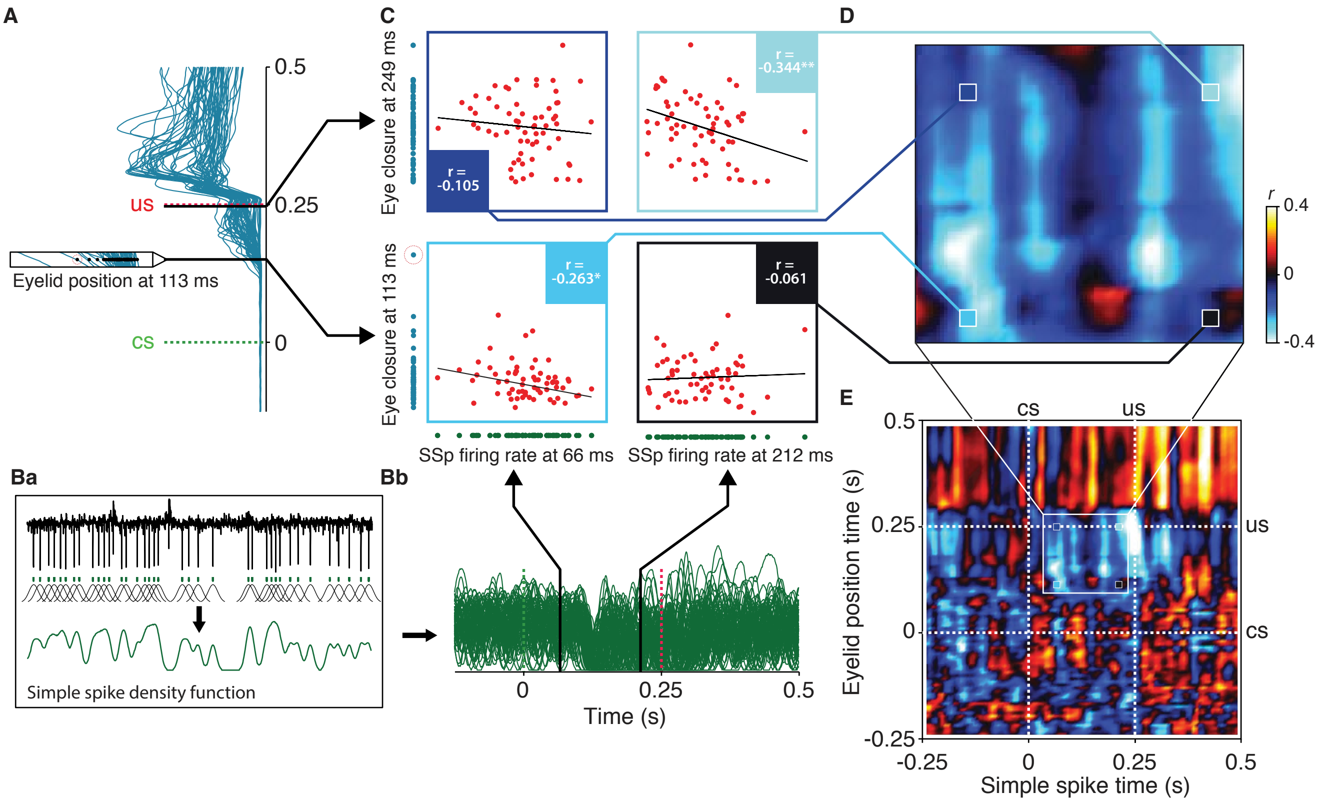

Figure S2. Construction of the simple spike-eyelid position correlation matrix, Related to Figure 2. (A) The y-axis of the eventual correlation matrix, E, denotes the time at which eyelid position data is taken. Here, eyelid traces for one recording are shown, rotated 90° counterclockwise. In the example at 113 ms after CS onset, the magnified window shows for each trial 10 data points, of which the means (black dots, red dashed circle indicates an outlier) are used for a correlation with spike activity. The same approach is taken to get eyelid position data at 249 ms. (Ba) To create a spike density function for each trial, Purkinje cell simple spike timestamps are convolved with a 41 ms wide Gaussian kernel, shown overlaid in black. The summated green trace provides a continuous measure of simple spike density. (Bb) The x-axis of the eventual correlation matrix, E, denotes the time at which simple spike activity is taken. Here, spike density traces for all trials of the same recording as in A are shown. Using the same approach outlined in A, 10-datapoint means are extracted for each trial at two example locations, here 66 and 212 ms after CS onset. (C) Between-trial correlations of eyelid position and simple spike activity data extracted at the example time-points. Simple spike activity at 66 ms correlates more strongly to eyelid position at 113 ms, than at 249 ms. Conversely, spike activity at 212 ms relates better to eyelid position at 249 ms than at 113 ms. If these correlations were done across different recordings, their trial data would be standardized before combining them. (D) The r-values for each of the example correlations translate to a color and find their way into the correlation matrix at the appropriate time points on the x- (spike) and y- (eyelid position) axis. (E) The resultant full correlation matrix shows the temporal dynamics of the correlation between simple spike activity and eyelid position. The current cell shows negative correlation throughout the ISI, with a minor early focal point, and a major late focal point.

Table S1. Summary of linear mixed-effects models, Related to Experimental Procedures.

**A.** For outcome variable **% Simple spike suppression** with random intercepts for **Purkinje cell ID**:				
**Fixed effect**	**Coefficient**	**F-test**	**P**	**Power***
Intercept	22.39 ± 2.53	$F_{1,972}$ = 78.03	<0.0001	**-**
CR (yes/no)	4.4 ± 1.14	$F_{1,972}$ = 14.87	0.00012	96.4%
**B.** For outcome variable **CR amplitude (% eyelid closure)** with random intercepts and slopes for **Purkinje cell ID**:				
**Fixed effect**	**Coefficient**	**F-test**	**P**	**Power**
Intercept	32.69 ± 2.47	$F_{1,699}$ = 174.51	<0.0001	-
% Simple spike suppression	0.334 ± 0.084	$F_{1,699}$ = 15.82	<0.0001	99%
**C.** For outcome variable **% Simple spike suppression** with random intercepts for **Purkinje cell ID**:				
**Fixed effect**	**Coefficient**	**F-test**	**P**	**Power**
Intercept	23.78 ± 2.3	$F_{1,972}$ = 107.3	<0.0001	-
CS-Complex spike (yes/no)	4.57 ± 1.14	$F_{1,972}$ = 16.2	<0.0001	86.2%
**D.** For outcome variable **CR amplitude (% eyelid closure)** with random intercepts for **Purkinje cell ID**:				
**Fixed effect**	**Coefficient**	**F-test**	**P**	**Power**
Intercept	37.98 ± 3.33	$F_{1,352}$ = 130.04	<0.0001	**-**
CS-Complex spike (yes/no)	5.20 ± 2.22	$F_{1,352}$ = 5.52	0.0194	83.8%
**E.** For outcome variable **CR amplitude (% eyelid closure)** with random intercepts for **Purkinje cell ID:**				
**Fixed effect**	**Coefficient**	**F-test**	**P**	**Power**
Intercept	65.28 ± 8.82	$F_{1,245}$ = 61.09	<0.0001	-
CS-Complex spike latency (ms)	-0.26 ± 0.10	$F_{1,245}$ = 8.74	0.0034	93.8%
**F.** For outcome variable **CS-Complex spike latency (ms)** with random intercepts for **Purkinje cell ID**:				
**Fixed effect**	**Coefficient**	**F-test**	**P**	**Power**
Intercept	91.47 ± 3.31	$F_{1,345}$ = 764.35	<0.0001	-
CR (yes/no)	-5.16 ± 1.29	$F_{1,345}$ = 15.99	<0.0001	98.2%
**G.** For outcome variable **CR amplitude (% eyelid closure)** with random intercepts for **Purkinje cell ID:**				
**Fixed effect**	**Coefficient**	**F-test**	**P**	**Power**
Intercept	29.24 ± 3.79	$F_{1,351}$ = 59.69	<0.0001	-
% Simple spike suppression	0.29 ± 0.06	$F_{1,351}$ = 24.48	<0.0001	99.8%
CS-Complex spike (yes/no)	3.08 ± 2.19	$F_{1,351}$ = 1.98	0.1604	-
**H.** For outcome variable **CR amplitude (% eyelid closure)** with random intercepts for **Purkinje cell ID**:				
**Fixed effect**	**Coefficient**	**F-test**	**P**	**Power**
Intercept	55.02 ± 8.8	$F_{1,246}$ = 38.83	<0.0001	-
% Simple spike suppression	0.24 ± 0.07	$F_{1,246}$ = 12.94	0.0004	94.6%
CS-Complex spike latency (ms)	-0.24 ± 0.09	$F_{1,246}$ = 7.65	0.0061	79.4%

Table S2. Summary of molecular layer interneuron recordings, Related to Figure 4.

Cell #	Hz	CV	CV2	# Valid Trials	Mean CR amplitude (%)	Mean CR onset (ms)	% CRs	% Spike facilitation in ISI	ISI Hz x CR amplitude r-value
1	6.6	0.69	0.66	25	68.1	105.6	88	673**	0.29
2	23.2	0.84	0.31	34	46.1	82.8	97.1	104**	-0.59***
3	17.8	0.84	0.58	8	53.5	91.3	100	78**	-0.24
4	8.4	0.74	0.62	10	30.7	104	100	233**	0.19
5	7.9	1.52	0.91	85	58.6	127.5	95.3	175**	0.34**
6	36.8	1.53	1.00	17	61.9	83.7	94.1	279**	0.73**
7	11.4	1.26	0.65	28	58.3	99.5	96.4	894**	-0.55**
8	26.3	0.82	0.53	15	65.6	98.2	100	192**	0.47
9	8.5	1.45	0.99	15	74.8	96.6	100	1048**	0.12
10	24.5	0.89	0.41	29	53.2	57.1	93.1	174**	0.73**
11	20.9	0.69	0.61	73	33.6	149.4	34.2	98**	0.41*
12	22.0	0.70	0.58	40	32.5	85.2	87.5	79**	0.63*
13	41.8	1.37	0.76	47	25.3	94.2	78.7	150**	0.40***
Mean	19.7	1.03	0.66	33	50.3	98.1	89.6	321.3	0.23

* $p < 0.05$, ** $p < 0.005$, *** $p < 0.001$
